# Characterization of the complete mitochondrial genome of *Diodon liturosus* with phylogenetic consideration

**DOI:** 10.1080/23802359.2017.1331321

**Published:** 2017-05-30

**Authors:** Jun Ma, Guang Hong, Chao He, Yan Chen, Huapu Chen, Hai Huang

**Affiliations:** a Hainan Key Laboratory for Conservation and Utilization of Tropical Marine Fishery Resources, College of Life Science and Ecology, Hainan Tropical Ocean University, Sanya, PR China;; b Fisheries College, Guangdong Ocean University, Zhanjiang, Guangdong, China

**Keywords:** Mitochondrial genome, *Diodon liturosus*, phylogenetic

## Abstract

In this study, the complete mitochondrial genome of *Diodon liturosus* was sequenced and analyzed. The mitochondrial genome is 16,512 bp long and consists of 13 protein-coding genes, two rRNA genes, 22 tRNA genes and a control region. The gene order and composition of *D. liturosus* mitochondrial genome was similar to that of most other vertebrates. The nucleotide composition of the light strand in descending order is 28.69% of A, 31.64% of C, 23.66% of T and 16% of G. The NADH dehydrogenase subunit 6 (ND6) and 8 tRNA genes were localized in the light strand, and all other mitochondrial genes were encoded on the heavy strand. The phylogenetic analysis by maximum-likelihood method revealed that *D. liturosus* has the closer relationship to the *Diodon holocanthus.*

The *Diodon liturosus* is a commercially important aquaculture fish species, which mainly distributes in the Indo-West Pacific region (Bogorodsky et al. 2011). With the aim of achieving to find new DNA markers for the studies on population genetics of *D*. *liturosus*, we determined to sequence the complete mitochondrial genome of *D. liturosus* using the next-generation sequencing (NGS) techniques strategy (Xie et al. 2015; Chen et al. 2016). *D. liturosus* was obtained in the South China Sea (N18°12′45.00″ E109°19′45.68″) at 5 June 2016 and the muscle of specimen was preserved in 95% ethanol until DNA isolation. The total genomic DNA was extracted by using salting-out procedure (Chen et al. 2016), and stored in Hainan Tropical Ocean University (Sanya, China) for the subsequent analysis.

The complete mitochondrial genome of *D. liturosus* (Genbank accession number KY682080) is 16,514 bp in length, consisting of 13 protein-coding genes, two ribosomal RNA genes (12S rRNA and 16S rRNA), 22 transfer RNA genes (tRNA) and one control region, which is similar with the typical vertebrates (Chen et al. 2015). Most of the genes are encoded on the heavy strand, with only the NADH dehydrogenase subunit 6 (ND6) and 8 tRNA genes [Gln, Ala, Asn, Cys, Try, Glu, Pro, Ser (GCT)] encoded on the light strand. Overall nucleotide compositions of the light strand are 28.40% of A, 30.71%of C, 24.79%of T and 16.11%of G. However, the most representative base is G and the bias against C was observed, which is different from the base compositions of mitochondrial genome of other teleosts.

There are two types of start codons and four types of stop codons. The two types of start codons are ATA (ND5) and ATG of the other 12 genes. Four types of stop codons are TAA (ND1, ATP8, ATP6, COX3, ND4L, ND5), TAG (ND2, ND3), AGA (COX1, COX2, CYTB) and AGG (ND4, ND6). The 12S and 16S rRNA genes are localized between the tRNA-Phe (GAA) and tRNA-Leu (TAA) genes. The 22 tRNA genes vary from 67 to 75 bp in length. All these could be folded into the typical cloverleaf secondary structure although numerous non-complementary and T–G base pairs exist in the stem regions. The control region was 864 bp in length, localized between tRNA-Pro (TGG) and tRNA-Phe (GAA) genes. The nucleotide composition of the control region was 31.48% of A, 21.06% of C, 16.32% of G, 31.13% of T.

The phylogenetic tree for *D. liturosus* was constructed with the complete mtDNA sequences from 13 species of Tetraodontiformes by using the maximum-likelihood methods. As shown in Figure 1, the *D. liturosus* was close to *Diodon holocanthus.* Thus this result supported the monophyly of *D. liturosus*.

**Figure 1.**
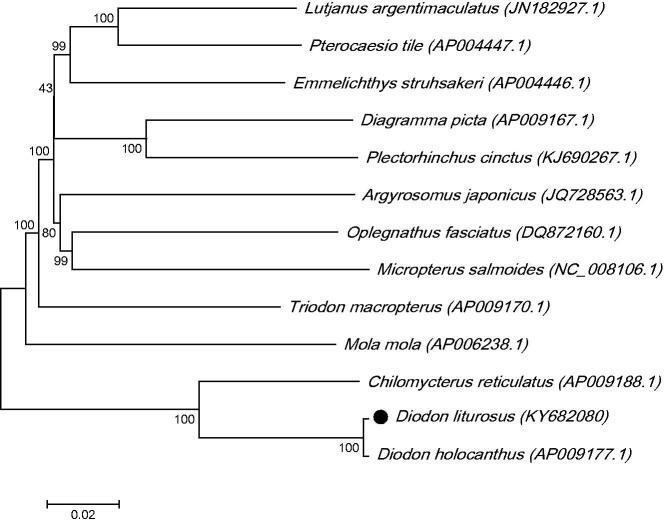
The maximum-likelihood phylogenetic tree of *Tetraodontiformes*. Numbers on each node are bootstrap values of 100 replicates.
